# MiR-423-5p prevents MALAT1-mediated proliferation and metastasis in prostate cancer

**DOI:** 10.1186/s13046-021-02233-w

**Published:** 2022-01-11

**Authors:** Carmela Ferri, Anna Di Biase, Marco Bocchetti, Silvia Zappavigna, Sarah Wagner, Pauline Le Vu, Amalia Luce, Alessia Maria Cossu, Jayakumar Vadakekolathu, Amanda Miles, David J. Boocock, Alex Robinson, Melanie Schwerdtfeger, Virginia Tirino, Federica Papaccio, Michele Caraglia, Tarik Regad, Vincenzo Desiderio

**Affiliations:** 1 Department of Precision Medicine, University of Campania “Luigi Vanvitelli”, Via L. De Crecchio, 7, 80138 Naples, Italy; 2 Medicina Futura Group, Coleman S.p.A, Via Alcide De Gasperi 107/109/111, 80011 Acerra, NA Italy; 3 The John van Geest Cancer Research Centre, School of Science and Technology, Nottingham Trent University, Clifton Lane, Nottingham, NG11 8NS UK; 4 Laboratory of Precision and Molecular Oncology, Biogem Scarl, Institute of Genetic Research, Contrada Camporeale, 83031 Ariano Irpino, Italy; 5 Clinical and Experimental Sciences, Faculty of Medicine, Southampton General Hospital, University of Southampton, Coxford Rd, Southampton, SO16 5YA UK; 6 Department of Life Sciences, Faculty of Health, Education and Life Sciences, Birmingham City University, Birmingham, B15 3TN UK; 7 Department of Experimental Medicine, University of Campania “Luigi Vanvitelli”, 80138 Naples, Italy; 8 Department of Medicine, Surgery and Dentistry “Scuola Medica Salernitana”, University of Salerno, Via S. Allende, 84081 Baronissi, Italy

**Keywords:** Prostate, Cancer, miRNAs, lncRNAs, miR-423-5p, Malat-1, Cellular biology, Molecular biology, Gene expression

## Abstract

**Background:**

The long non-coding RNA (lncRNA), MALAT1, plays a key role in the development of different cancers, and its expression is associated with worse prognosis in patients. However, its mechanism of action and its regulation are not well known in prostate cancer (PCa). A general mechanism of action of lncRNAs is their interaction with other epigenetic regulators including microRNAs (miRNAs).

**Methods:**

Using lentiviral stable miRNA transfection together with cell biology functional assays and gene expression/target analysis, we investigated the interaction between MALAT1 and miR-423-5p, defined as a target with in silico prediction analysis, in PCa.

**Results:**

Through bioinformatic analysis of data available from TCGA, we have found that MALAT1 expression correlates with high Gleason grade, metastasis occurrence, and reduced survival in PCa patients. These findings were validated on a TMA of PCa showing a significant correlation between MALAT1 expression with both stage and grading. We report that, in PCa cells, MALAT1 expression and activity is regulated by miR-423-5p that binds MALAT1, downregulates its expression and inhibits its activity in promoting proliferation, migration, and invasion. Using NanoString analysis, we unraveled downstream cell pathways that were affected by miR-423-5p expression and MALAT1 downregulation and identified several alterations in genes that are involved in metastatic response and angiogenic pathways. In addition, we showed that the overexpression of miR-423-5p increases survival and decreases metastases formation in a xenograft mouse model.

**Conclusions:**

We provide evidence on the role of MALAT1 in PCa tumorigenesis and progression. Also, we identify a direct interaction between miR-423-5p and MALAT1, which results in the suppression of MALAT1 action in PCa.

**Supplementary Information:**

The online version contains supplementary material available at 10.1186/s13046-021-02233-w.

## Background

Long Non-coding RNAs (lncRNAs) play important roles in the regulation of several physiological processes and diseases. Their function is based upon the control of expression of genes involved in development, differentiation, metabolism, and diseases [1–7]. MALAT1 (Metastasis Associated Lung Adenocarcinoma Transcript *1)* is a lncRNA highly expressed by several types of cancers and associated to cancer progression and worse prognosis in cancer patients [8–12]. MALAT1 promotes cell proliferation and migration, and its depletion inhibits both in vitro cell motility and metastasis in mouse cancer models [13–17]. In prostate cancer (PCa), MALAT1 plays a role in tumorigenesis and cancer progression and has been proposed as a potential therapeutic target for castration resistant PCa [18]. Although MALAT1 expression and role in cancer are widely described in the literature, the mechanisms regulating its activity are not well known.

MicroRNAs (miRNAs) are small non-coding RNAs that regulate a wide variety of cell processes primarily by preventing the translation of target mRNAs [19]. In cancer, miRNAs can function as either tumour suppressors or oncogenes by promoting or preventing the expression of factors involved in proliferation, invasion, and metastasis [20, 21]. Besides these functions, the interactions between miRNAs and LncRNAs have been reported [22]. LncRNAs were shown to act as miRNAs’ decoys or sponges, or compete with miRNAs for binding to shared target mRNAs [23]. For instance, MALAT1 promotes PCa progression through its interaction with and inhibition of miR-140, resulting in an increase of the apoptotic inhibitory factor, BIRC6 [24]. On this light, we used two online available tools Targetscan and miRanda to search for additional MALAT1 targets and identified MiR-423-5p as a possible interactor. MiR-423-5p plays different roles in the tumorigenesis and progression of glioblastomas, hepatocellular carcinoma, colon, gastric, and ovarian cancers [25–28]. However, its role in PCa and its relationship with MALAT1, are unknown. Based on this background, we hypothesized that *miR-423-5p* could interact with *MALAT1* to regulate/alter its function in PCa. We reveal that *miR-423-5p* directly interacts with *MALAT1* and downregulates its expression in prostate cancer cells. Moreover, we demonstrate that the overexpression of miR-423-5p inhibits MALAT1-mediated proliferation, migration, and invasion of PCa cells. Using NanoString technology, we also evaluated the expression of 770 genes involved in each step of PCa progression, including angiogenesis, extracellular matrix remodelling (ECM), epithelial-to-mesenchymal transition (EMT), and metastasis. We found that MALAT1 downregulation by miR-423-5p negatively affects the metastatic and angiogenic pathways.

These results were confirmed by in vivo experiments showing that *miR-423-5p* inhibits *MALAT1*-mediated tumour growth and metastasis. Collectively, these results demonstrate that *miR-423-5p* prevents PCa progression through the inhibition of MALAT1.

## Methods

### Cell lines and growth conditions

DU145 (ATCC® HTB-81™) and PC3 (ATCC® CRL-1435™) prostate cancer cell lines were purchased from ATCC and cultured in EMEM (BE12-6621, Lonza) and F-12 k Nut Mix (1x) (21127-022, Gibco Life Technologies) respectively. The LNCaP (ATCC® CRL-1740™) prostate cancer cell line was also purchased from ATCC and cultured in RPMI-1640 (11530586, Gibco Life Technologies). The media were supplemented with 10% (v/v) foetal calf serum (FCS) and 1% (w/v) L-glutamine (Lonza). Cells were incubated at 37 °C in 5% (v/v) CO_2_ and 100% (v/v) humidity.

### Generation of PC3-LUC2 clones

Firefly luciferase (LUC2) expression was induced in PC3 pLKO and PC3 mir-423 cells using the pcDNA3.1/LUC2 plasmid. The plasmid was isolated from glycerol stock using the Plasmid Midi Kit (QIAGEN), according to manufacturer’s protocol and the concentration was measured using a Nanodrop. Plasmid was immediately stored at − 20 °C. Cells were transfected at 50-60% confluency using the Lipofectamine 3000 kit (Invitrogen) by mixing 7.5 μL of Lipofectamine 3000 reagent in 250 μL Opti-MEM with 10 μL of P3000 reagent in 250 μL Opti-MEM and 5 μg of pcDNA3.1/LUC2 plasmid. After 30 min of incubation at room temperature, 250uL of the mix was added per well (6-well plate). Cells were selected with 500 μg/mL G418. The expression of LUC2 gene was confirmed using the IVIS® imaging system. D-luciferin at a concentration of 150 μg/mL was added to the cell suspension 10 min before the acquisition of the bioluminescent signal intensity (BLI). Clones from single cells were generated and those with the highest LUC2 expression were selected.

### Retroviral expression of empty vector and miR-423-5p-mimic

PC3, DU145 and LNCaP prostate cancer cell lines were seeded in a 96 multiwell, 35,000 cells/ well, and treated with Sh MIMIC Lenti miR-423-5p lentiviral particles (GE Healthcare Dharmacon V1SMHS_000254) at 2.5, 5, 10 and 20 MOI. Control empty backbone cells were obtained using lentiviral particles generated from the pLKO.1 Empty plasmid (MISSION® pLKO.1-puro Empty Vector Control Plasmid DNA Sigma Aldrich SHC001). Hexadimethrine Bromide (SIGMA H9268-5G) was used in complete growth media for each cell line to facilitate the infection. After 16 h incubation, infection media was removed and substituted with complete growth medium for each cell line. Puromycin (INVIVOGEN) was then added to each cell complete growth medium at a final concentration of 1 μg/ml for the selection. The lentiviral particles used to infect DU145, PC3 and LNCaP were produced according to the manufacturer’s recommendations. The lentiviral packaging mix was also purchased from Sigma (SHP001).

### Proliferation assay

pLKO.1 Empty and miR-423-5p PC3 and LNCaP clones were assessed for proliferation using CyQuant NF fluorescent dye (Thermo Fisher Scientific Cat n. C35007) according to the manufacturer protocol. Twenty-five thousand cells in replicates for each clone were seeded in 96 MW and the fluorescence intensity was measured after 24 and 48 h for each sample using a fluorescence microplate reader (TECAN 200) with excitation at 485 nm and emission detection at 530 nm.

### Wound healing assay

Wound Healing assay was performed to evaluate migration: PC3 and LNCaP pLKO.1 Empty and miR-423-5p clones were seeded in a 24 multiwell in replicates. At approximately 80-90% confluence a scratch was performed in the middle of the wells using a 10 uL sterile tip. Wound Area was then recorded at T0, 12 h and 24 h.

### Migration and invasion assay

Cultrex® BME Cell Invasion Assay (Trevigen Cat n.3455-096-K) 96 plates were used to investigate PC3 and LNCaP pLKO.1 Empty and miR-423-5p migration and invasion capability according to the manufacturer protocol. Empty chambers were used to assess migration and BME matrix coated chambers were used for invasion. After 48 h the assay chamber fluorescence was measured using a fluorescence microplate reader (TECAN) at 485 nm excitation, 520 nm emission.

## Transwell migration and invasion assay

For the transwell assay, 5 × 10^4^ PC3 pLKO.1 Empty and miR-423-5p clones were seeded in serum-free medium into the upper Boyden chamber (Sarstedt, Germany) uncoated and coated with 0.2 mg/mL Matrigel (Corning, Life Sciences, NY, USA), and then 600 μL of  F-12 k Nut Mix supplemented with 10% FBS was added into the lower chamber. After 48 h, each transwell chamber was washed with PBS buffer and the non-migrated and non-invasive cells were carefully removed using cotton swabs. Then, the cells were fixed in 100% methanol and stained with 0.5% (v/v) crystal violet (Pro-Lab Diagnostics, Canada) in 70% ethanol (Fisher Scientific™, UK). The images were obtained using an inverted microscope. Crystal violet salts were dissolved with 30% acetic acid (v/v) in dH2O and the absorbance measured at 540 nm (CLARIOstar® Plus, BMG LABTECH, Germany).

## Quantitative RT-PCR

RNA extraction from PC3, DU145 and LNCaP pLKO.1 Empty and miR-423-5p clones was performed using RNeasy Mini Kit and miRNeasy micro kit (Qiagen Ref. 74106, 217084) according to the manufacturer protocol. For miR423-5p, TaqMan Micro RNA Reverse Transcription Kit was used to obtain cDNA together with specific retrotranscription primers for miR-423-5p (Applied Biosistems Ref: 4427975) and U6 (Applied Biosistems Ref. 4427975) as housekeeping gene. RT-qPCR was performed using TaqMan Universal PCR Master Mix (Applied Biosistems Ref. 4304437) and specific qPCR primers for miR-423-5p (Applied Biosistems Ref. 4427975) and U6 (Applied Biosistems Ref: 4427975) were used. For Malat1, VEGFB, PTTG1, SNRPF, COL1A1, AGR2, CXCL8, NR3C1, LOX, E-cadherin, N-cadherin, Fibronectin-1 and ZEB-1 the PROMEGA Reverse Transcription System (Ref. A3500) was used to obtain cDNA. RT-qPCR was performed using iTaq Universal SYBR Green Supermix (BioRad Cat.No. 172-5124) and specific qPCR primers for Malat1 FW:GGATCCTAGACCAGCATGCC RV:AAAGGTTACCATAAGTAAGTTCCAGAAAA), VEGFB (FW: AGTGCTGTGAAGCCAGAC RV: GGAGTGGGATGGGTGATG), PTTG1 (FW: CTGTAAAGACCAAGGGACCCCT RV: GCAGGAACAGAGCTTTTTGCTT), SNRPF (FW: AGAGTAGCCTGCAACATTCG RV: GATAGCCCTTGTACTCCATTCC), COL1A1 (FW: GAGGGCCAAGACGAAGACATC RV: CAGATCACGTCATCGCACAAC), AGR2 (FW: ATGAGTGCCCACACAGTCAA RV: GGACATACTGGCCATCAGGA), CXCL8 (FW: GACAGCAGAGCACACAAGC RV: GGCAAAACTGCACCTTCAC), NR3C1 (FW: ACAGCATCCCTTTCTCAACAG RV: AGATCCTTGGCACCTATTCCAAT), LOX (FW: CACAGGACATCATGCGTATGC RV: CCACTTCAGAACACCAGGCAC), E-cadherin (FW: AGTGGGCACAGATGGTGTGA RV: TAGGTGGAGTCCCAGGCGTA), N-cadherin (FW: TCG ATT GGT TTG ACC ACG G RV: GAC GGT TCG CCA TCC AGA C), Fibronectin-1, (FW: GGAGGAAGCCGAGGTTTTAAC RV: ACGCTCATAAGTGTCACCCA), ZEB-1 (FW: GCACAACCAAGTGCAGAAGA RV: GCCTGGTTCAGGAGAAGATG) and GAPDH (FW:GTCTCCTCTGACTTCAACAGCG RV:ACCACCCTGTTGCTGTAGCCAA) as housekeeping gene (Eurofins MWG Operon, Sigma-Aldrich). The results were then analyzed using 2–∆∆Ct method.

## In situ hybridization in PCa TMA

Tissue Slides were deparaffinized with subsequent washes in Xylene, 100% Ethanol, 90% Ethanol, 70% Ethanol, PBS, 3% Hydrogen peroxidase in methanol and dH2O. Pre-Hybridization was conducted by slides incubation with Hybridization Solution (Exiqon). 100 nM of custom LNA™ detection probe for MALAT1 (/5BiosG/ATGCTACTCTTCTAAGTCTTCA, Exiqon) was added to fresh Hybridization buffer 1X, heated at 95 °C for 4 min, and incubated on the slides at 54 °C overnight (in humidifying chamber). Subsequently, the tissue slides were washed with 5x, 1x and 0.02X SSC (saline-sodium citrate buffer) at 54 °C and after quickly with PBS. 2.5% horse serum was used for blocking, then slides were washed with Avidin D solution and then Biotin. The primary antibody was then added (Anti-Biotin Mouse for Malat1, Exiqon) and slides incubated at 4 °C overnight. Pan Specific antibody was used as secondary antibody while ABC Vectastain kit and DAB kit were used to visible stain the RNAs. Mayer’s Haematoxylin was used to visible stain the cell nuclei. The slides were then paraffinized, scanned and sent to the histopathologist for expression scoring.

### Luciferase assay

#### Recombinant pmirGLO Dual Luciferase Plasmids production

To clone our target sequence Binding site A and B into a plasmid (pmirGLO Dual-Luciferase miRNA Target Expression Vector - Promega E1330), we synthesized two partially complementary oligoes for each with 4 nt overhangs compatible for cloning into the vector plasmid paying careful attention to the 5′ > 3′ orientations. When annealed, oligonucleotides form double stranded DNA with overhangs for cloning into Xbal site in pmirGLO vector. We used this protocol for obtain 3 different recombinant plasmids: pmirGLO-MALAT1 plasmid for binding site A, pmirGLO-MALAT1 plasmid for binding site B and also pmirGLO-MALAT1 plasmid with mutated binding sequence (negative control). The steps are explained below in detail.

1. Annealing and cloning procedure:
  Anneal each pair of oligonucleotides: 1 μl oligo Fw (100 μM) + 1 μl oligo Rev. (100 μM) + 8 μL H2O for 10 μL total. Anneal in a thermocycler at 95 °C 5 min and then leave on the bench at RT to cool down for 1 h. Dilute the annealed oligonucleotides 1:200. The oligo sequences used are shown in Table 1.

**Table 1:** Primers used for recombinant pmirGLO Dual Luciferase Plasmids production

MALAT1 Binding site A Forward	MALAT1 Binding site A Reverse
CTAGAGAAGCCTCAGCTCGCCTGAAGGCAGGTCCCCTCTGACGCCTCCGGGAGCCCAGT	CTAGACTGGGCTCCCGGAGGCGTCAGAGGGGACCTGCCTTCAGGCGAGCTGAGGCTTCT
MALAT1 Binding site B Forward	MALAT1 Binding site B Reverse
CTAGACCTTTTTTTAAGATTTTTCAGGTACCCCTCACTAAAGGCACCGAAT	CTAGATTCGGTGCCTTTAGTGAGGGGTACCTGAAAAATCTTAAAAAAAGGT
MALAT1 Mutated Binding site Forward	MALAT1 Mutated Binding site Reverse
CTAGAGAAGCCTCAGCTCGCCTGAAGGCATTTTTTTTGACGCCTCCGGGA	CTAGATCCCGGAGGCGTCAAAAAAAAATGCCTTCAGGCGAGCTGAGGCTTC

Primers used for recombinant pmirGLO Dual Luciferase Plasmids production
  Digestion vector reaction: linearize the pmirGLO vector with the appropriate restriction enzymes to generate overhangs that are complementary to the annealed oligonucleotide overhangs. 1 μg pmirGLO vector + 2 μl 10X RE Buffer + 1 μl Xbal (Promega- R6181) + Nuclease Free water to final volume of 20 μL. Incubate the digestion reaction at 37oC for at least 1 h. Run ~ 200 ng on agarose gel to ensure complete digestion. When digest is complete, heat inactivate at 65 °C for 20 min.
  Ligation reaction: 100 ng pmirGLO Xbal digested vector + 2 μl annealed oligonucleotides duplex from step 1 (1:200 dilution) + 2 μl 10x DNA ligase buffer + 1 μl T4 ligase (T4 DNA Ligase- New England Biolabs) + Nuclease Free water to 20 μl final volume. Incubate the ligation reaction at 16 °C for 30 min and then at 65 °C for 10 min.
2. Transformation

The pmirGLO-MALAT1 Dual Luciferase Plasmids obtained in the previous step were amplified individually in competent cells. First, the cells were thawed on ice for 5 min and mixed before transferring 50 μL to a chilled sterile polypropylene culture tube. Twenty-five nanograms of pmirGLO-MALAT1 plasmid was added to the competent cells and placed on ice for 10 min. Cells were heat-shocked at 42 °C for 45 s, placed on ice for 2 min, added to 450 μL of lysogeny broth (LB; Sigma-Aldrich) containing 100 μg/mL ampicillin (Sigma-Aldrich) and were shaken at 37 C° for 60 min. All 500 μL was plated on ampicillin containing LB-Agar plates overnight, and afterwards clones were isolated and shaken overnight at 37 °C in 5 mL of LB broth containing ampicillin. Plasmid was extracted using the QIAprep® Miniprep (QIAGEN). Cells were pelleted in a centrifuge and resuspended in 250 μL of Buffer P1. Two hundred fifty microliters of Buffer P2 was added and mixed by inverting the tube, and then 350 μL of neutralization solution (N3) was added and mixed by inverting the tube. Cells were centrifuged for 10 min at 14,000 x g. The supernatant was transferred to a QIAprep 2.0 spin column and centrifuged for 1 min at 12,000 x g. The flow through was discarded and two washes of 500 μL wash solution was added and the column centrifuged for 1 min at 12,000 x g each time. The plasmid was eluted from the column by adding 50 μL of elution buffer, waiting 2 min and centrifuging the columns at t 12,000 x g for 2 min. The concentration of the plasmid was then measured using a NanoDrop ND1000.

### Dual luciferase assay

The Dual-Luciferase Reporter Assay System (Promega) was used to detect firefly and renilla luciferase from the pmirGLO-MALAT1 A/B plasmids and pmirGLO-MALAT1 Mut (mutated site) plasmid in Mimic miR-423-5p LNCaP stable cell line or LNCaP pLKO.1-puro (empty vector). Twenty-four hours after transduction with recombinant plasmids (separately) using Lipofectamine 3000, the cells were washed with PBS in preparation for lysis. Cells were no more than 95% confluent at the time of lysis. One hundred microliters of Passive lysis Buffer was added to each well and the plate was rocked gently for 15 min. One hundred microliters of Luciferase Assay Reagent II was added in each well and after 15 min the measurement for firefly luciferase activity was done with Luminometer Infinite M200 Pro TECAN. Then, 100 μL of Stop & Glo reagent was added and renilla luciferase activity was measured after 15 min. Each sample was read twice, and luciferase activity (firefly luciferase / renilla luciferase) was normalized to the LNCaP pLKO.1-puro (empty vector).

### Proximity ligation assay

PLA assay was performed using the Duolink® In Situ Red Starter Kit Mouse/Rabbit (DUO92101, Sigma-Aldrich, St Louis, MO, USA) and following the manufacturer recommendations.

Cells were washed once with phosphate-buffered saline (PBS) and fixed in 4% formaldehyde (v/v) in PBS on ice for 30 min. The samples were permeabilized with 70% Ethanol (v/v) in Nuclease Free Water overnight at + 4 °C and washed three times with PBS. The samples were then pre-hybridized with Hybridization solution 2X (Exiqon) for 6 h at 54 °C (in humidify chamber). One hundred nanometers of specific oligonucleotide probes (Custom LNA™ detection probe for MALAT1 and miRCURY LNA miRNA Detection Probe, Exiqon) were added to fresh Hybridization buffer 1X, heated at 95 °C for 4 min, and incubated on fixed/permeabilized cells at 54 °C overnight (in humidify chamber). Subsequently, the cells were washed with 0.02X SSC (saline-sodium citrate buffer) for 1 h at 54 °C and after quickly with PBS. The samples were incubated with 2/3 drops of blocking solution (Duolink® PLA, Sigma-Alderich) for 1 h at 37 °C (in humidify-chamber) and then with the appropriate primary antibodies in Diluent Antibody solution (Duolink® PLA, Sigma-Alderich) at 4 °C overnight. The probe solution was prepared by diluting the corresponding species-specific minus PLA probe and plus PLA probe (Duolink® PLA, Sigma-Alderich) 1:5 into Duolink Antibody Diluent. After two washes with 1X Wash buffer A, the coverslips were transferred to a prewarmed humidified chamber and incubated with the probe solution for 1 h at 37 °C.

The subsequent PLA ligation and amplification steps were performed according to the manufacturer’s instructions (Duolink® PLA, Sigma-Alderich). Briefly, the PLA probe solution was aspirated from the cells and washed twice with wash buffer A for 5 min each under gentle agitation. Cells were incubated with freshly prepared ligation mix for 30 min at 37 °C. Samples were then washed twice with wash buffer A for 5 min each with gentle agitation and incubated with freshly prepared amplification mix for 100 min at 37 °C. To enhance the signal intensity, the amplification step can be extended to 2–4 h if needed. Cells were washed twice with wash buffer B for 10 min with gentle agitation and a final wash with 0.01× wash buffer B for 1 min. Coverslips were mounted onto glass slides using Duolink In Situ Mounting Medium with DAPI. Images were captured by Leica TCS SP5 fluorescent microsystems using appropriate filters. All images were taken with the same exposure time and the same threshold to allow subsequent quantitative analysis in the respective channel. At least 50 nuclei per sample were imaged and the images were processed with ImageJ. Experiments were repeated three times.

### NanoString nCounter gene expression analysis

A total of 730 cancer-related human genes and 40 internal reference genes were contained on cancer pathway panel reporter and capture probe. For analysis, LNCaP pLKO.1-puro and LNCaP Mimic miR-423-5p cells were used. One hundred nanograms of total RNA was used for hybridization, and analyzed on an nCounter Digital Analyzer (NanoString Technologies, Inc., Seattle, WA) according to the manufacturer’s instructions. Briefly, a hybridization reaction was set up at room temperature for each sample with the following components: 3 μL of Reporter CodeSet, 5 μL of hybridization buffer, up to 5 μL (100 ng) of sample RNA, and 2 μL of Capture ProbeSet. Briefly the tubes were spined down and immediately placed in the pre-heated 65 °C thermal cycler for at least 16 h. When the hybridization time is up, two of the 96 well plates were centrifuged at 670 g for 2 min brake off and the Prep Station machine was initiated. Data quality control was implied using nSolver analysis software (NanoString Technologies). Reporter counts were normalized to each sample using positive control and housekeeping genes. The mean values were shown to fold change or log2 transformation.

### Gene ontology (GO) analysis

Metascape.org was used to enrich genes for GO biological processes and pathways.

The software identified all statistically enriched terms (GO Biological Processes, GO Cellular Components and GO Molecular Functions), accumulative hypergeometric *p*-values and enrichment factors were calculated and used for filtering. Remaining significant terms were then hierarchically clustered into a tree based on Kappa-statistical similarities among their gene memberships. Then 0.3 kappa score was applied as the threshold to cast the tree into term clusters.

### Tumour transplantation assay

Non-obese diabetic/severe combined immunodeficiency (NOD/SCID) mice were acquired (Charles River, U.K.) and kept in accordance with the Animals (Scientific Procedures) Act 1986. The mice were used for tumour transplantation Assay (Xenotransplantation assay) by subcutaneous injection of PC3 control (PC3 Control/Luc2) or PC3 miR-423-5p-mimic (PC3 miR-423-5p-mimic/Luc2 (3 × 10^6^ suspended in 100ul PBS). Tumours’ growth and metastases’ formation were monitored using a Perkin Elmer™ bioluminescence-based in vivo imager.

### Bioinformatic and statistical analyses

In silico validation was performed by analysing the gene expression of MALAT1 with clinical parameters using the following datasets: Lapointe (*n* = 26) [29], Tomlins (*n* = 75) [30], Varambally (*n* = 19) [31] and The Cancer Genome Atlas (TCGA) (*n* = 281) [32]. The gene expression profiles were generated using gene expression microarrays (Lapointe, Tomlins and Varambally) and RNA-sequencing (TCGA). The gene expression of MALAT1 was selected and min-max normalised across the complete patient population within each dataset. Gene expression of MALAT1 across healthy, primary, and metastatic prostate cancer was compared using datasets of Lapointe, Tomlins and Varambally. A comparison of MALAT1 expression wit Gleason scoring (GS6, GS7, GS8 and GS9 + 10) and disease-recurrence was performed using the TCGA dataset. Unpaired t-test was used to analyse differences between the sample groups, comparing disease status and Gleason score. Kaplan-Meier curves were generated presenting disease-free survival (DFS) in relation to MALAT1 expression. Patient groups were separated according to median expression and survival curves were analysed using Mantel-Cox test. *P*-values below 0.05 were considered to be significant (*p* ≤ 0.05 = *, *p* ≤ 0.01 = **, *p* ≤ 0.001 = *** and *p* ≤ 0.0001 = ****). Analysis was performed using GraphPad Prism 8. The in silico identification miR-423-5p binding sites on MALAT-1 was analysed using TargetScan (http://www.targetscan.org/vert_71/).

For TMA analysis all data are presented as the mean ± SEM for at least three independent experiments. For each experiment, the statistical tests are indicated in the Results section. The student’s t-test was conducted using Prism 8 (Graphpad Software, La Jolla, CA, USA). Significant (‘*’/*p* = < 0.05), very significant (‘**’/*p* = < 0.01), highly significant (‘***’/*p* = < 0.001) or very highly significant (‘****’/*p* = < 0.0001). Spearman’s Rho bivariate analysis and X squared test of contingency were performed using IBM SPSS Ver. 25, *p*-value < 0.05 is considered statistically significant.

Other statistical analyses were performed using the GraphPad Prism 8 student *t*-test and Anova.

## Results

### High level of MALAT1 expression correlates with decreased survival of PCa patients

**Fig. 1:**
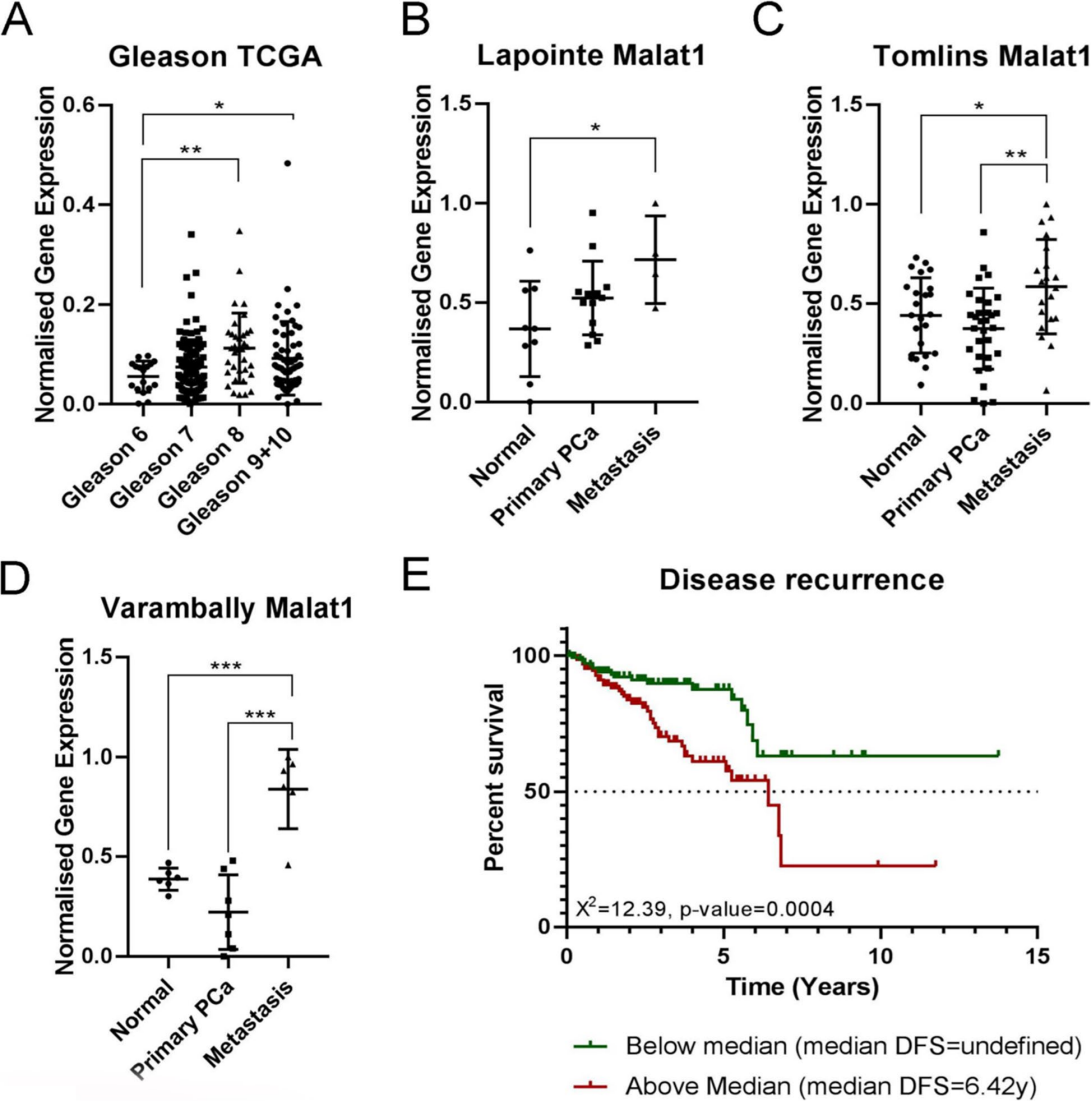
MALAT1 expression correlates with advanced and metastatic prostate cancer, and reduced patients’ survival. **A** Graph representing the correlation between MALAT-1 expression and prostate cancer Gleason grade using bioinformatic analysis of the TCGA database. **B**-**D** graphs representing the correlation between MALAT-1 expression and prostate cancer metastasis using bioinformatic analyses of the Lapointe, Tomlins, and Varambally microarrays databases. *P ≤ 0.05, **p ≤ 0.01, ***p ≤ 0.001 and ****p ≤ 0.0001. **E** Cox regression analysis of the correlation between MALAT1 expression and survival of prostate cancer patients using the TCGA database. X2 = 12.39, *p* = 0.0004

To investigate *MALAT1* expression level in PCa and its correlation with patients’ Gleason stage and survival, we performed a bioinformatic analyses of data derived from TCGA, Lapointe, Tomlins, and Varambally microarrays databases (Fig. 1A, B, C and D). The analyses showed that *MALAT1* gene expression correlates with higher PCa Gleason grade (Fig. 1A) and is significantly increased in advanced and metastatic PCa (Fig. 1B, C and D). Moreover, the association between *MALAT-1* expression and cancer-specific survival was also investigated using the TCGA database. In this analysis, the capacity of high or low expression of *MALAT1* to predict clinical outcome was assessed. High expression of *MALAT1* was associated with a significant reduction in cancer-specific survival (*χ*^2^ = 12.39, *p* = 0.0004) over a 14-year timing, when compared to patients with low *MALAT1* expression (Fig. 1E). In addition, we have investigated the relationship of *MALAT1* expression with both TNM staging and WHO grading by using a commercially available TMA of PCa. We found that *MALAT1* expression in cancer tissues is significantly higher than in normal tissues (*P* = 0.001). The percentage of high positive cells (to MALAT1) correlates with TNM staging. Indeed, in T stage III/IV *MALAT1* was significantly higher than in T stage II/III and its expression is associated to the presence of nodal (N1) and distant (M1) metastases. Moreover, it also correlates with high WHO grading (in grade 3-4 was higher than in grade 1-2). All these differences were statistically significant (Table 2 and supplementary fig. 1). These results demonstrate that *MALAT1* expression correlates with metastases and worse survival in PCa patients.

**Table 2:** Correlation of clinic-pathological features with MALAT1 expression in PCa TMA corhort

	Cases	Negative	Low Positive	High Positive	pValue (X^**2**^)
	(Tot 71)				
**Normal**	8–11.3%	7–87.5%	1–12.5%	0–0%	0.001
**Cancer**	**63**–88.7%	12–19%	37–58.7%	14–22.2%	
**T1-T2**	30–47.6%	6–20%	23–76.7%	1–3.3%	0.006
**T3-T4**	33–52.4%	6–18.2%	14–42.4%	13–39.4%	
**N0**	51–80.9%	11–21.6%	34–66.7%	6–11.7%	0.001
**N1**	12–19.1%	1–8.3%	3–25%	8–66.7%	
**M0**	55–87.3	11–20%	35–63.7%	9–16.3%	0.001
**M1**	8–12.7%	1–12.5%	2–25%	5–62.5%	
**Stage 1-2**	30–47.6%	6–20%	23–76.7%	1–3.3%	0.001
**Stage 3-4**	33–52.4%	6–18.2%	14–42.4%	13–39.4%	
**WHO Grade 1-2**	24–38.1%	6–25%	17–70.8%	1–4.2%	0.001
**WHO Grade 3-4**	37–58.7%	5–13.5%	19–51.4%	13–35.1%	

Spearman’s Rho bivariate analysis correlationcoefficient indicates a positive correlation between Malat1 histopathological score and patient’s clinical parameters analyzed. Correlations are significant at the 0.01 and 0.05 levels (2-tailed). The X squared tests of contingency performed between the same factors is significant with a *p*-value < 0.01. Frequency tables and all the statistical analyses were performed using IBM SPSS Ver. 25

### MiR-423-5p interacts with MALAT-1 and downregulates its expression in PCa cells

**Fig. 2:**
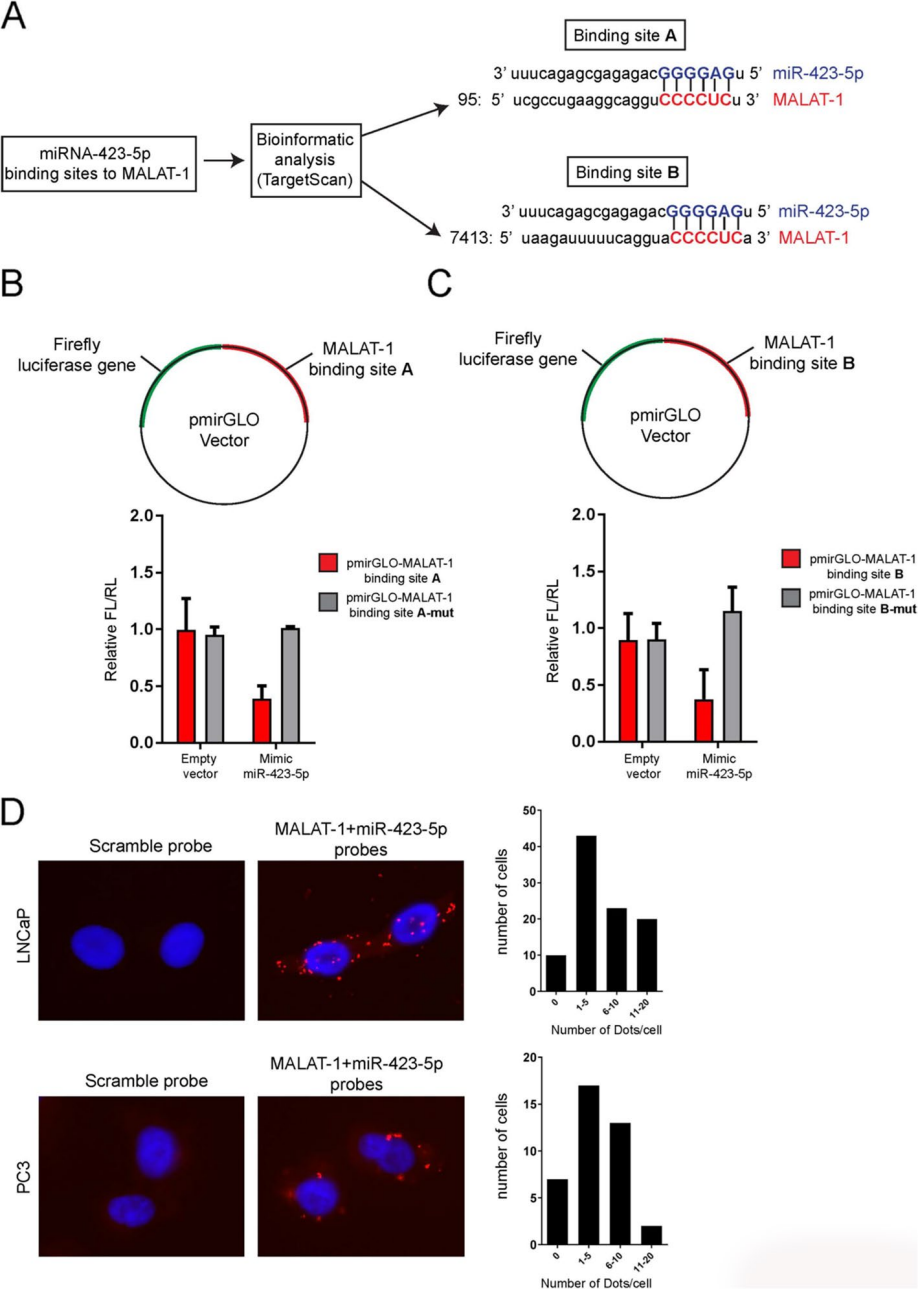
MiR-423-5p interacts with MALAT1 in prostate cancer cell lines. **A** Schematic representation of the bioinformatic method (TargetScan) used to identify miRNA-423-5p binding sites on the MALAT1. **B**, **C** Plasmids used to generate Firefly luciferase expressing vectors under the control of unmutated and mutated miRNA-423-5p binding sites on the MALAT1 (Upper panels). Graphs representing the relative expression of FL/RL using vectors under the control of unmutated and mutated miRNA-423-5p binding sites on the MALAT1 (Lower panels). **D** Representative images of MALAT1 and miR-423-5p expression in the prostate cancer cell lines LNCaP and PC3 using the proximity ligation assay (PLA) (Left panels). Graphs representing the number of dots that were detected by PLA in LNCaP and PC3 cells

To identify *MALAT1* interacting miRNAs, a TargetScan bioinformatic analysis was performed (Fig. 1F). The analysis identified *miR-423-5p* as a potential *MALAT1*-interacting miRNA and highlighted the presence of two potential *miR-423-5p* binding sites at position 95 and 7413 of the *MALAT1* 5′-3′ sequence (Fig. 2A). To confirm this results in silico, the two binding sequences, and their corresponding mutants, were respectively inserted into the multiple cloning site (MCS) of the pmirGlo Dual Luciferase miRNA target Expression Vector. These constructs (pmirGLO-MALAT1 binding site A and pmirGLO-MALAT1 binding site B) enable the evaluation of *miRNA-423-5p* activity through the firefly luciferase reporter gene. The generated constructs were then transfected into LNCaP PCa cells, in the presence or absence of the miRNA-423-5p mimic (Fig. 2B and C).

When miRNA-423-5p mimic was present, we observed a reduction in the expression of the firefly luciferase gene with both constructs. However, this effect was absent with the constructs that carried mutations in the binding sequences (Fig. 2B and C). Furthermore, the interaction between endogenous *MALAT-1* and *miR-423-5p* was additionally confirmed using a proximity ligation assay (Fig. 2D). In fact, the assay results in a fluorescent signal in the form of a red spot when *MALAT-1* and *miR-423-5p* are closer than 40 nm and this effect occurred in both LNCaP and PC3 PCa cell lines (Fig. 2D).

**Fig. 3:**
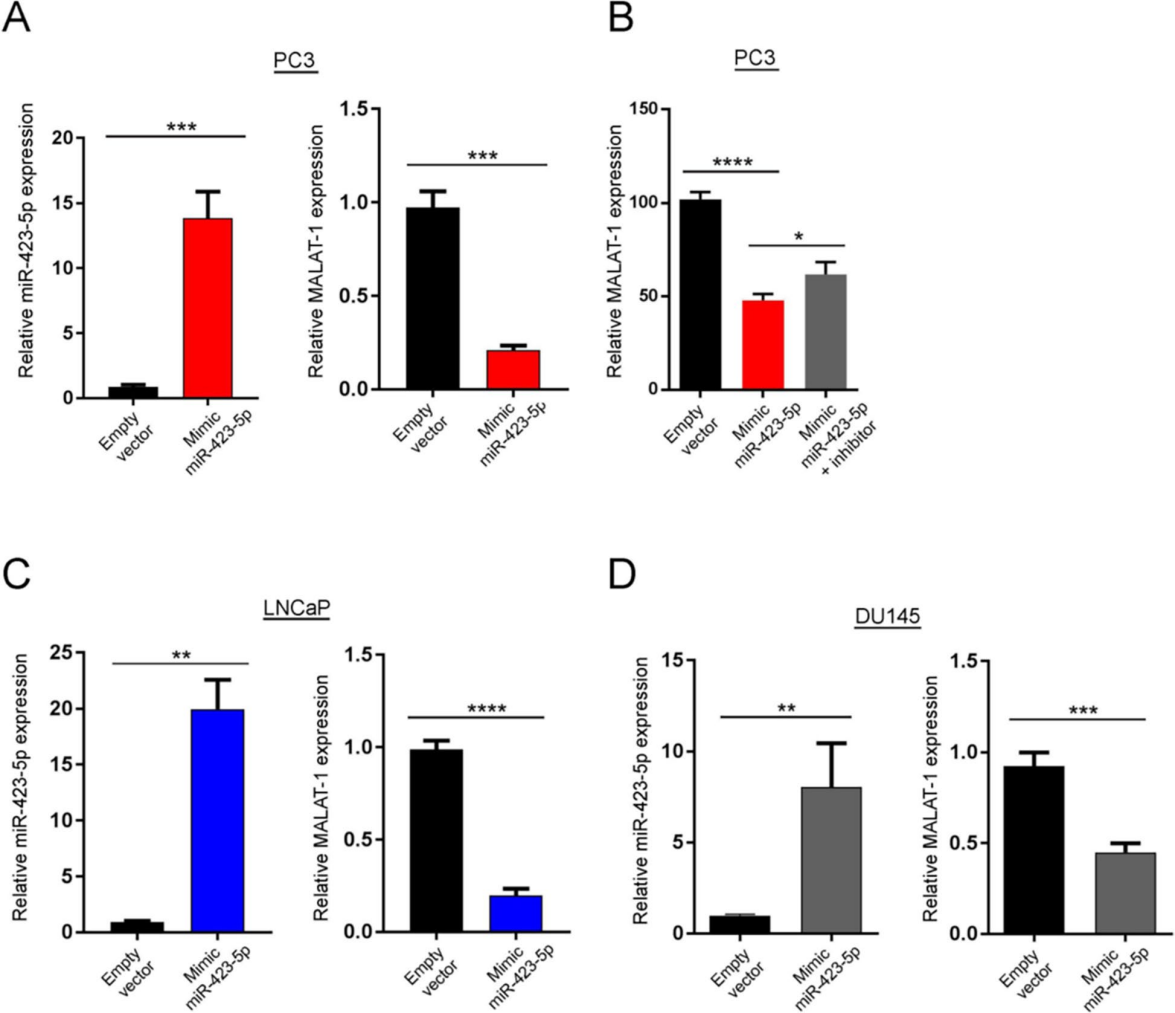
MiR-423-5p expression downregulates the expression of MALAT1 in prostate cancer cell lines. **A**-**D** Graphs representing the effect of expressing miR-423-5p mimic on the relative MALAT1 expression in PC3, LNCaP and DU145 prostate cancer cells. ***P = 0.0004, ***p = 0.001, **p = 0.0023, ****p < 0.0001, **p = 0.0071 and ***p = 0.0009. **B** Graph representing the effect of expressing miR-423-5p mimic on the relative MALAT1 expression in PC3 that is rescued using an inhibitor of miR-423-5p. ****P = 0.0120 and *p < 0.0001

To investigate the effect of *miR-423-5p* binding to *MALAT1*, the relative expression level of *MALAT1* is assessed after lentiviral expression of *miR-423-5p* mimic in PC3, LNCaP and DU145 PCa cells (Fig. 3A). Using real time PCR (RT-qPCR), we observe that *MALAT1* expression is significantly decreased in cells expressing *miR-423-5p* mimic when compared to the controls (Fig. 3A, C and D). We also record that this effect is reversed in PC3 cells that express the inhibitor of the *miR-423-5p* (Fig. 3B). These results demonstrate that *miR-423-5p* binds *MALAT1* and negatively regulates its expression in PCa cells.

### MiR-423-5p prevents MALAT1-mediated proliferation and metastasis of PCa cells

**Fig. 4:**
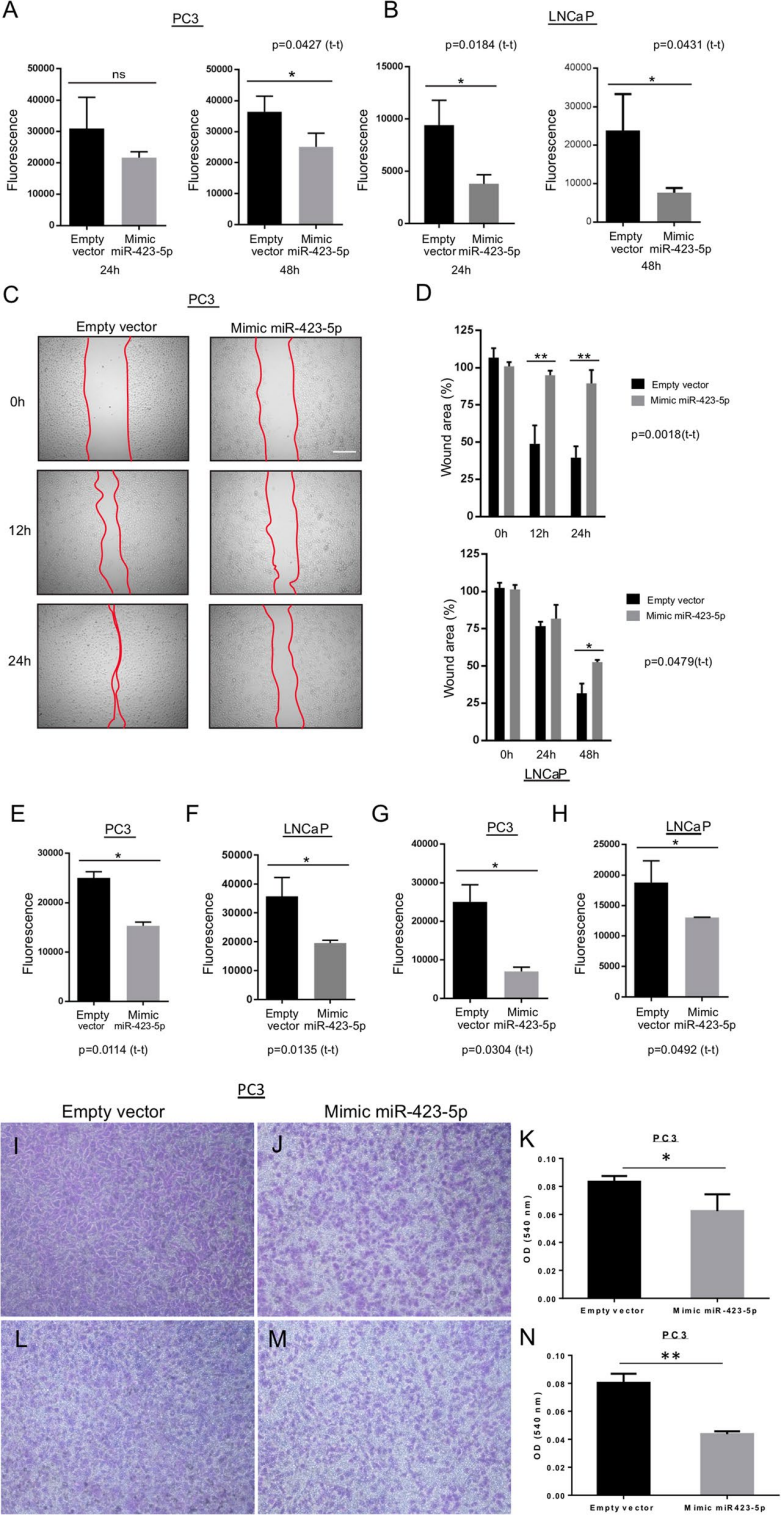
Effect of miR-423-5p mimic expression on cell proliferation, migration, and invasion of PC3 and LNCaP prostate cancer cell lines. **A**, **B** Graphs representing the effect of miR-423-5p mimic expression on cell proliferation of PC3 and LNCaP prostate cancer cells at 24 and 48 h. P = ns (24 h), *p = 0.0427 (48 h), *p = 0.0184 (24 h) and *p = 0.0431 (48 h). **C**, **D** Graphs representing the effect of miR-423-5p mimic expression on cell migration of PC3 and LNCaP prostate cancer cells using the scratch assay. **P = 0.0018 and *p = 0.00479. **E**, **F** Graphs representing the effect of miR-423-5p mimic expression on cell migration of PC3 and LNCaP prostate cancer cells using the transwell assay. *P = 0.0114 and *p = 0.0135. **G**, **H** Graphs representing the effect of miR-423-5p mimic expression on cell invasion of PC3 and LNCaP prostate cancer cells using the transwell invasion assay. *P = 0.0304 and *p = 0.0492. Representative images of migration in Boyden chambers of **I** (empty vector) and **J** miR-423-5p mimic expressing PC3 cells after 48 h and crystal violet labeling. **K** Graphs representing the effect of miR-423-5p mimic expression on cell migration of PC3 cells. * *P* = 0.049. Representative images of invasion in Boyden chambers of **L** (empty vector) and **M** miR-423-5p mimic expressing PC3 cells after 48 h and crystal violet labeling. **N** Graphs representing the effect of miR-423-5p mimic expression on cell invasion of PC3 cells. ** *P* = 0.006

We investigated whether inhibition of *MALAT1* expression induced by *miR-423-5p* affects its functions in PCa cell proliferation and invasion. We show that the proliferation of PC3 and LNCaP PCa cells expressing *miR-423-5p mimic* is significantly decreased compared to controls (Fig. 4A and B).

Using the scratch assay, we have investigated the effects of the expression of *miR-423-5p mimic* on cell migration of PC3 and LNCaP cells and found that their migration is significantly decreased compared to the controls (Fig. 4C and D). We have confirmed these results using a transwell migration assay through Cultrex™ Cell Invasion and Migration Assay (Fig. 4E and F). To investigate whether cell invasion is affected, we have used a transwell invasion assay, and showed that there is a significant decrease in the invasive abilities of PC3 and LNCaP cells that expressed *miR-423-5p* mimic compared to controls (Fig. 4G and H). All these data have been also confirmed with a conventional Boyden Chamber assay on PC3 PCa cells and the data are shown in Fig. 4I-N. The overexpression of *miR-423-5p* inhibits *MALAT1*-mediated proliferation, migration, and invasion of prostate cancer cells.

### NanoString nCounter gene expression assay reveals the reduction of the metastatic response pathway in miR-423-5p-transduced prostate cancer cells

**Fig. 5:**
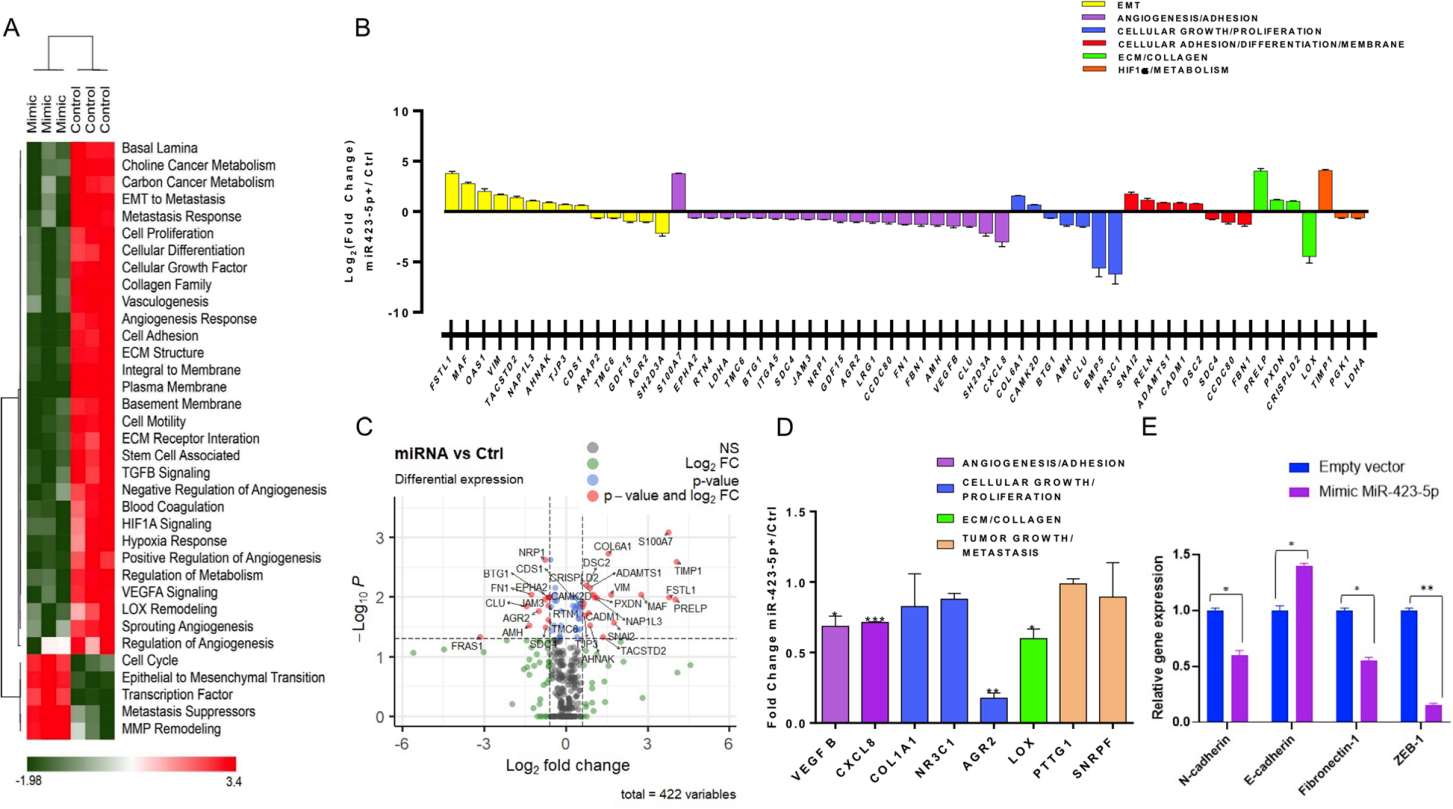
**A** High-level overview of altered pathways in miR-423-5p mimic compared to control samples. For each pathway, scores were generated using a linear combination (a weighted average) of gene expression values that represent pathways using nSolver Advance analysis module V 4.0. The pathways are listed on the horizontal axis and samples are listed vertically. The green colour indicates low scores; the red colour indicates high scores. Scores are displayed on the same scale via Z-transformation. Clustering of the scores were performed using unsupervised hierarchical clustering (Euclidean distance, complete linkage) and visualized using a Morpheus (Broad Institute, MA, USA). **B** Histogram graph of differentially expressed (DE) genes (down−/up-regulated) involved in Pan Cancer Progression pathways that were analysed by NanoString Technologies. Data are shown as Log2 fold compared to control (cells transduced with the empty vector). **C** Volcano plot showing differential expression of key genes upon miRNA KD in comparison to the control group. Y–axis represent corrected pValue (Benjamini–Yekutieli). The X-axis represents log2 fold change. The vertical dotted line indicates absolute fold change of 2 and the horizontal line denotes a corrected p- value of 0.05. Significant genes are labelled and colour-coded as red. **D** Histogram graph of differentially expressed (DE) genes validated by qPCR in PC3 prostate cancer cells. *P = 0.0263 (VEGF B), ***p = 0.0002 (CXCL8), **p = 0.0011 (AGR2), *p = 0.0143 (LOX). **E** Histogram graph of expression of genes involved in EMT

The roles *of MALAT1* in multiple physiological processes have been previously reported [1–7] and several findings indicated that it has also a close association with the expression of metastasis-associated genes [14–17]. PCa cell lines that were transduced with *miR-423-5p*-mimic showed low levels of MALAT1 compared to control. To dissect the role of the *miR-423-5p/MALAT-1* interaction in the context of PCa, we used NanoString technology to evaluate the genes or gene clusters that are modulated following *miR-423-5p*-mimic expression. This technology allows the use of function-specific mRNAs panels for simultaneous evaluations of cellular pathways and processes. On this light, we have used the PanCancer Progression Panel to perform multiplex gene expression analysis with 770 genes that are involved in each step of cancer progression, including angiogenesis, extracellular matrix remodeling (ECM), epithelial-to-mesenchymal transition (EMT) and metastasis [33]. From the analysis of the results a reduction of the metastatic response of the cells transduced with mimic *miR-423-5p* is recorded (Fig. 5A, B). Some of these genes have been additionally validated by quantitative real time PCR. We have found that several oncogenes are downregulated such as Vascular endothelial growth factor (VEGF) B, AGR2 that is associated with tumour growth and metastasis [34, 35], CXCL8, which drives angiogenesis and metastasis in several cancers including also PCa [36] and LOX (Lysyl oxidase), which is highly expressed in invasive tumours, and closely associated with metastases and poor patient clinical outcome [37] (Fig. 5D). On the basis of the data obtained by NanoString, we investigated on EMT pathway perturbation. We have found an increase of the E-Cadherin and a significant decrease of the Fibronectin-1, ZEB-1 and N-Cadherin gene expression (Fig. 5E). These data are in agree with a strong perturbation of the EMT induced by miR-423-5p that could be at least in part responsible for the effects on metastases of prostate cancer cells in vivo. These data suggest a role of *miR-423-5p* in inhibiting metastases and angiogenesis of PCa cells.

### In vivo effects of miR-423-5p on MALAT1-mediated tumor growth

**Fig. 6:**
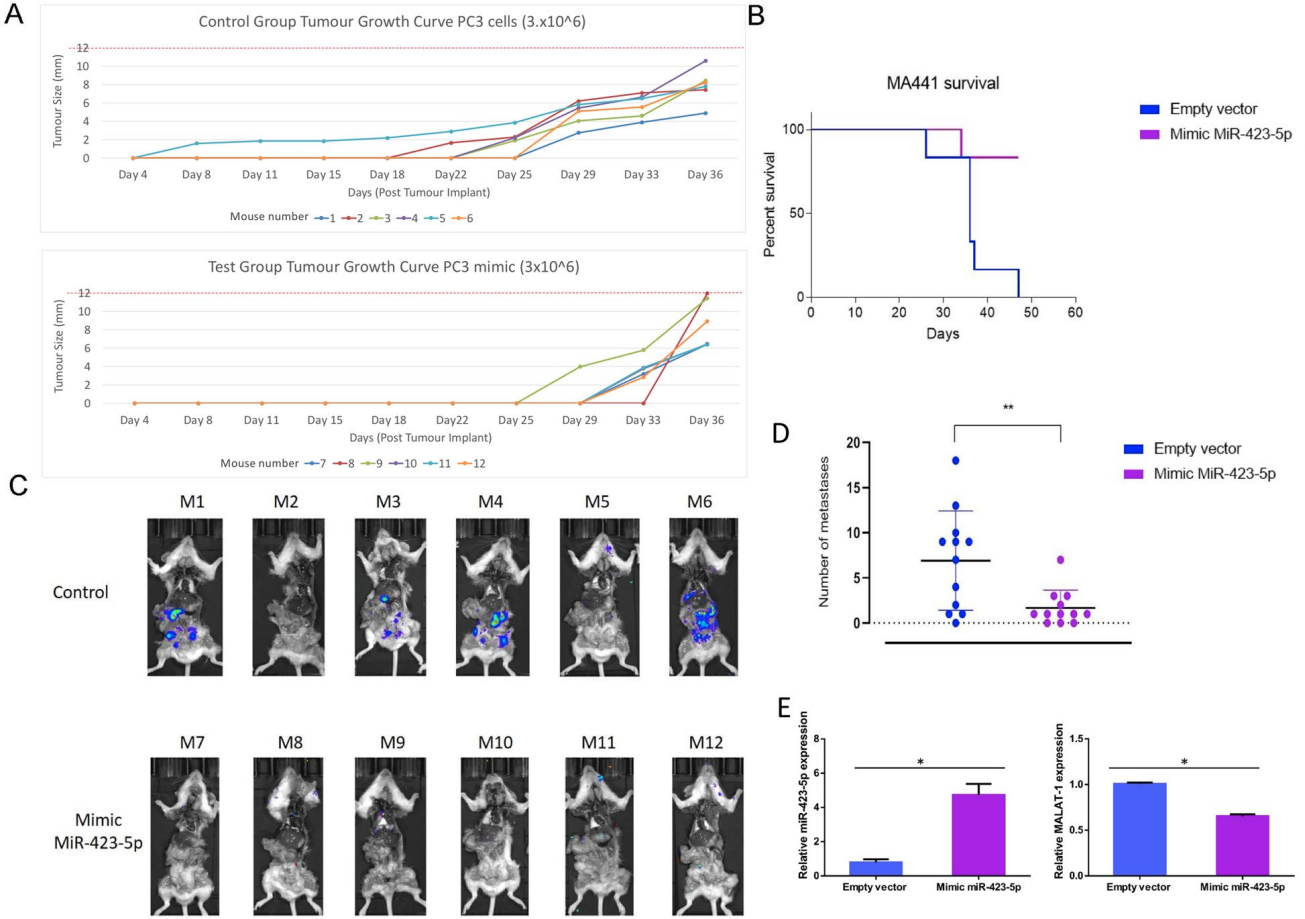
In vivo effect of MiR-423-5p mimic expression on survival and metastasis. **A** Graphs representing tumours’ formation and growth vs. time (Days) in PC3 control and MiR-423-5p mimic expressing cells that were xenografted NOD/SCID mice, respectively. **B** Kaplan-Mayer graph representing the percentage of survival NOD/SCID mice carrying PC3 cells xenografted control and MiR-423-5p mimic expressing tumours. **C** Representative images of metastases in the control and MiR-423-5p mimic groups (images from only one experiment shown, left panel). **D** Quantification of the number of metastasis is shown on the right panel graph (Total number of mice in each group = 12 from two independent experiments). **P = 0.0051. **E** Graphs representing the relative MALAT-1 and MiR-423-5p expression in the tumor tissue from control and MiR-423-5p mimic groups (*p < 0.05)

To validate our finding in vivo, we investigated the effects of *MiR-423-5p* mimic expression on mice survival, tumour growth and metastatic formation in a PC3 cells’ xenograft mouse model. *MiR-423-5p* mimic expressing PC3 cells have been stably transduced with a Luciferase expressing vector (Luc pcDNA) and subcutaneously injected into NOD/SCID mice. Tumours’ growth and metastases have been monitored using a bioluminescence-based in vivo imager. Surgically removed tumor tissues from nude mice of both control and *miR-423-5p* mimic group, at the end of the in vivo experiment, and graphical representation of tumor weight were shown in additional fig. 2. In the control group, the tumours appear at earlier times compared to the *miR-423-5p* mimic expressing xenografts (Fig. 6A), while mice in the *miR-423-5p* mimic expressing xenografts have a prolonged survival if compared to the control (Fig. 6B). Additionally, the number of metastases is significantly higher in the control group compared to that one in *MiR-423-5p* mimic expressing xenograft group (Fig. 6C and D). The reduced expression of MALAT1 in *MiR-423-5p* mimic group was validated by quantitative real time PCR (Fig. 6E). Taken together, these results suggest that *MiR-423-5p* inhibits the proliferation, invasion, and metastasis of PCa through mechanisms that involve the suppression of *MALAT-1* expression by *MiR-423-5p* itself.

## Discussion

*MALAT-1* is a lncRNA that plays important roles in the regulation of several physiological processes and diseases. Its expression has been associated with cancer progression and worse prognosis in cancer patients [8–12], which is reflected by its ability to promote cancer cells’ proliferation, migration, invasion, and metastasis [13–17]. *MALAT-1* has been shown to play a role in tumorigenesis and cancer progression of castration resistant prostate cancer [18]; on the other hand, its association with disease progression and survival in PCa patients is still unclear. Using bioinformatic tools, we showed that *MALAT-1* expression correlates with advanced and metastatic PCa and is associated with a decrease of PCa patients’ survival. In addition to the bioinformatic analysis, we have investigated the relationship of MALAT1 expression with TNM staging and WHO grading by using a commercially available TMA of PCa. We have found that the expression level of MALAT1 in cancer tissues is significantly higher than that in normal tissues and is associated to the presence of both lymph node and distant metastases and low grade of differentiation. Therefore, we show, for the first time, that MALAT-1 has potential diagnostic and prognostic values in PCa. Moreover, we investigated potential mechanisms that regulate *MALAT-1* expression and functions in PCa in order to highlight possible strategies of intervention based upon *MALAT-1* targeting in PCa. On this light, using in silico tools, we have identified *miR-423-5p* as a potential *MALAT-1* binding partner. *MiR-423-5p* belongs to the family of small non-coding RNAs regulating a wide variety of cellular processes [19] and playing different roles in the tumorigenesis and progression of glioblastomas, hepatocellular carcinoma, colon, gastric, and ovarian cancers [25, 26, 28, 38, 39]. Several studies have identified miR-423 as a diagnostic and prognostic biomarker in various tumors. In this light, our group has reported that *miR-423-5p* is increased after sorafenib treatment in serum of HCC patients responding to sorafenib treatment acting as surrogate marker of response [38]. Moreover, its overexpression promotes autophagy in HCC, inhibits proliferation and invasion in osteosarcoma, and reduces metastasis in glioblastoma [40, 41]. However, its role in PCa is still unknown.

In this study, we show that *MiR-423-5p* acts as a tumour suppressor through its regulation of *MALAT-1* oncogenic functions. Generally, LncRNAs limit miRNAs action by acting as miRNAs’ decoys or sponges, or by competing with miRNAs for binding to shared target mRNAs [23]. Conversely, we show that *miR-423-5p* reduces *MALAT-1* expression by binding to two specific regions of *MALAT-1,* resulting in the inhibition of its ability to promote cancer cells’ proliferation, invasion, and metastases in vitro and in vivo. Although, some of these effects may also be associated with mechanisms that are exclusively *miR-423-5p-*dependent*,* our results suggest that most of them are associated to *MALAT-1* downregulation*.* Indeed, recent studies reported the critical and specific function of *MALAT1* in regulating the expression of several target genes that are associated with metastases (e.g., *DRD1*, *COL6A1*, *STC1*) or that regulate metastatic formation (e.g., *GPC6*, *MCAM, PRKCE*) [15]. Moreover, MALAT1 is overexpressed in PCa tissues and cell lines, and its silencing impairs proliferation, migration, and invasion [24].

These data and interpretations are additionally supported by our Nanostring analysis that shows alterations of gene signatures that are related to metastasis and angiogenesis. These results are additionally validated by our in vivo experiments that clearly show that *miR423-5p* overexpression induces a strong and significant delay in the growth of the PCa xenografts that is paralleled by a significant decrease in both number and size of metastases. Overall, our findings demonstrate that *miR-423-5p* acts as a tumour suppressor by preventing prostate cancer cells’ proliferation, invasion and metastasis through mechanisms that involve, at least in part, the repression of *MALAT-1* expression and functions. Finally, we also suggest that miR-423-5p can be used in the development of targeting strategies for the treatment of patients with aggressive prostate cancer.

## Conclusions

In conclusion, we have demonstrated that *MALAT-1* expression is associated with both progression and survival of PCa patients. We identify *miR423-5p* as a new *MALAT-1* interactor suppressing its expression and function in PCa. These results suggest that *miR423-5p/MALAT-1* interaction has a strong relevance in PCa and should be further exploited for the designs of new therapeutic strategies.

## Supplementary Information

**Additional file 1: Additional figure 1.** The panel reported shows PCa patients tissues stained for Malat-1 by ISH. After DAB staining and reparaffinization, slides were scanned using the Hamamatzu Nanozoomer slide scanner (Nottingham City Hospital, Nottingham, UK) and sent to the histopathologist for the expression scoring. A and B show Malat-1 high positive Prostate Cancer tissue examples while B and C show Malat-1 low positive and negative Prostate Cancer tissue examples respectively. All the tissues photos were taken at 20x magnification using NDP view software version 2.

**Additional file 2: Additional figure 2.** Surgically removed tumor tissues from nude mice of both control and miR-423-5p mimic group, at the end of the in vivo experiment and graphical representation of tumor weight in the different groups. Data are presented as the mean ± SD (*n* = 6, *p < 0.05).

## Data Availability

All data generated or analysed during this study are included in this published article [and its supplementary information files].

## Abbreviations

AGR2: Anterior Gradient 2
BIRC6: Baculoviral IAP Repeat Containing 6
COL6A1: Collagen type VI alpha 1 chain.
CXCL8: C-X-C Motif Chemokine Ligand 8
DRD1: Dopamine Receptor D1
ECM: Extracellular matrix remodeling
EMT: Epithelial-to-mesenchymal transition
FN1: Fibronectin 1
GPC6: Glypican 6
HCC: Hepatocellular Carcinoma
MCAM: Melanoma Cell Adhesion Molecule
PCa: Prostate Cancer
PRKCE: Protein kinase C epsilon type
STC1: Stanniocalcin 1
TCGA: The Cancer Genome Atlas
VEGF: Vascular endothelial growth factor
