# Supplementary figures and images for: MiR-423-5p prevents MALAT1-mediated proliferation and metastasis in prostate cancer
Source: J Exp Clin Cancer Res. 2022 Jan 11;41:20. doi: 10.1186/s13046-021-02233-w (PMC8751098; doi:10.1186/s13046-021-02233-w)

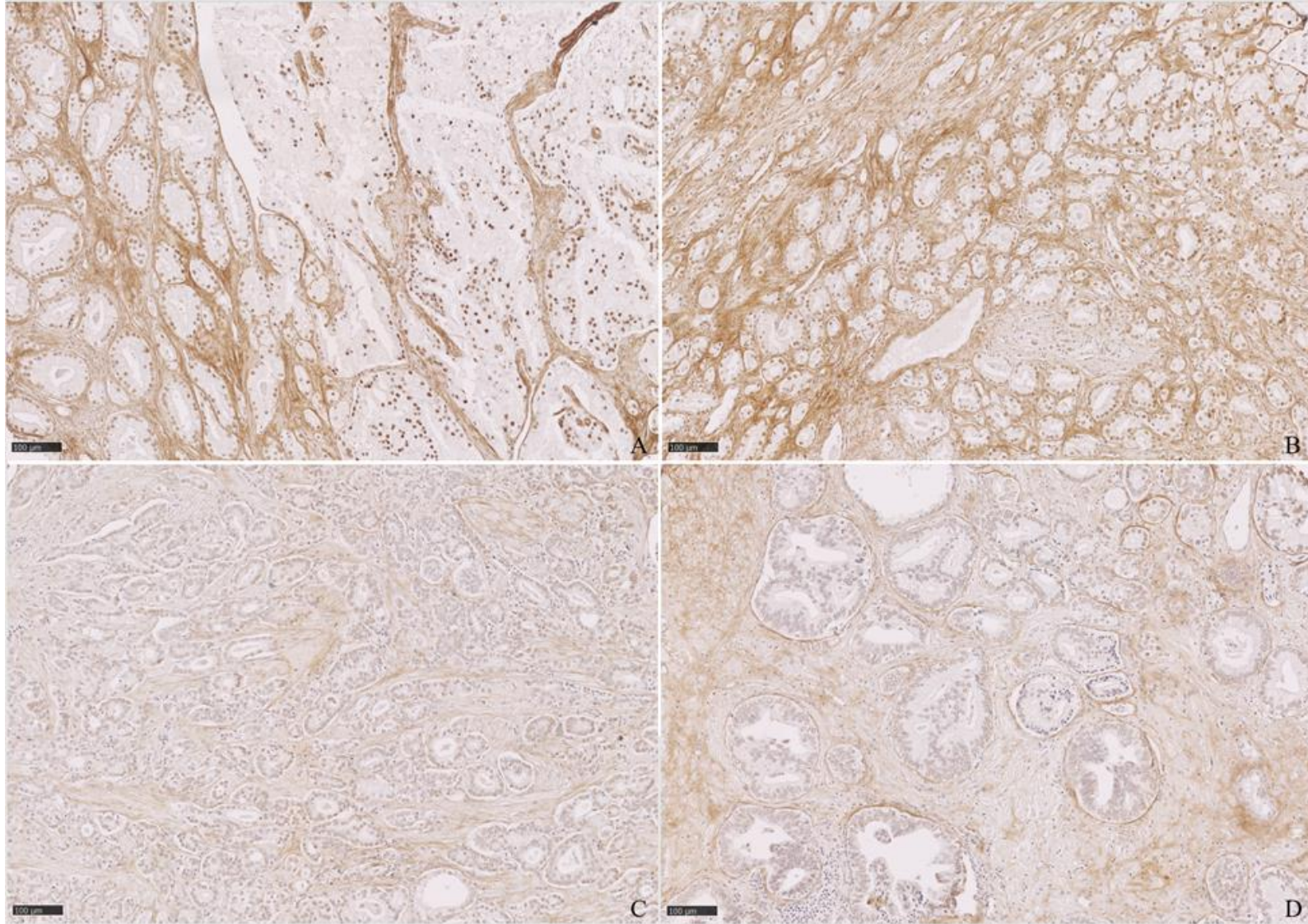

Supplement: Supplementary file 1 — Additional file 1: Additional figure 1. The panel reported shows PCa patients tissues stained for Malat-1 by ISH. After DAB staining and reparaffinization, slides were scanned using the Hamamatzu Nanozoomer slide scanner (Nottingham City Hospital, Nottingham, UK) and sent to the histopathologist for the expression scoring. A and B show Malat-1 high positive Prostate Cancer tissue examples while B and C show Malat-1 low positive and negative Prostate Cancer tissue examples respectively. All the tissues photos were taken at 20x magnification using NDP view software version 2. [file 13046_2021_2233_MOESM1_ESM.pdf]

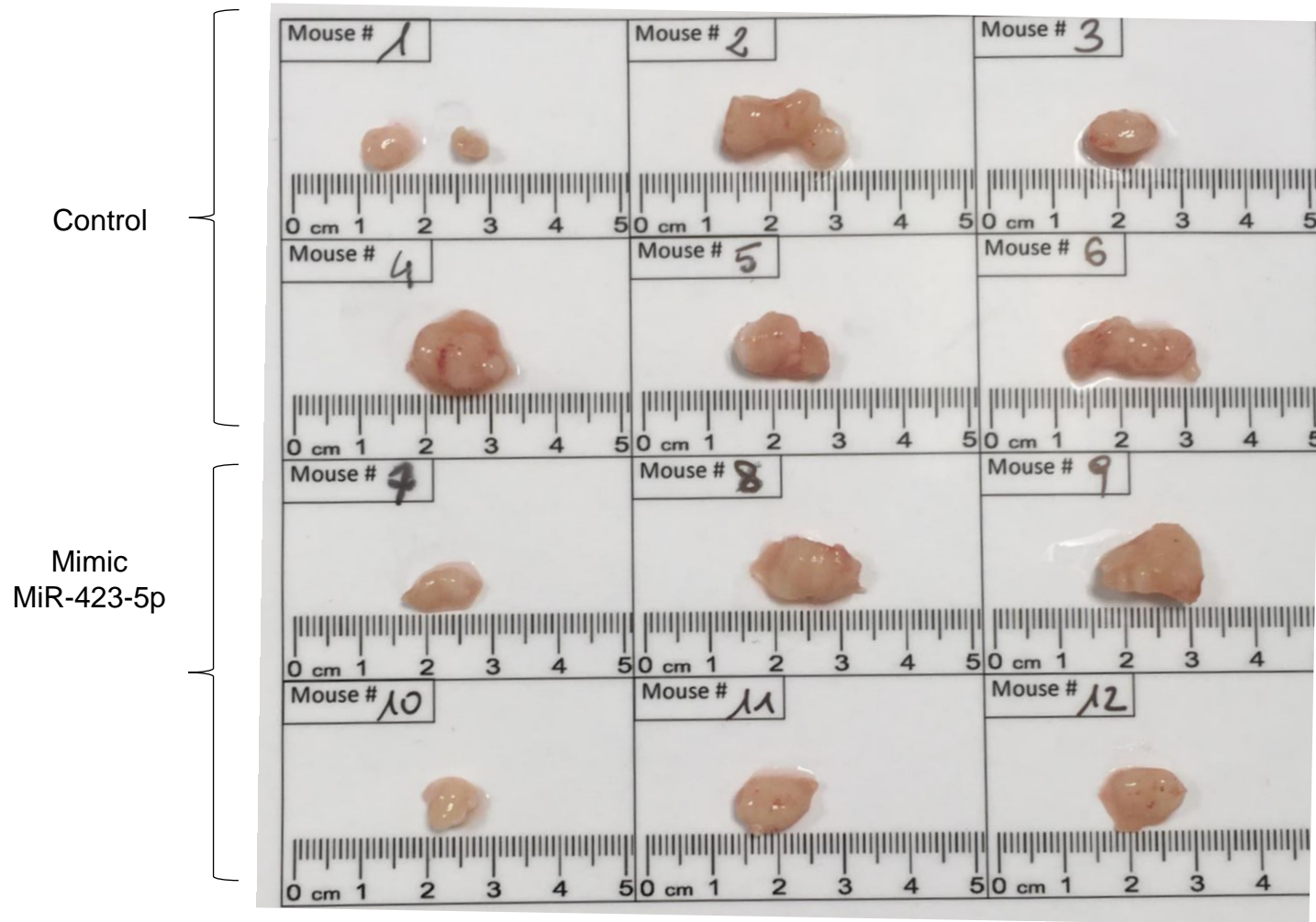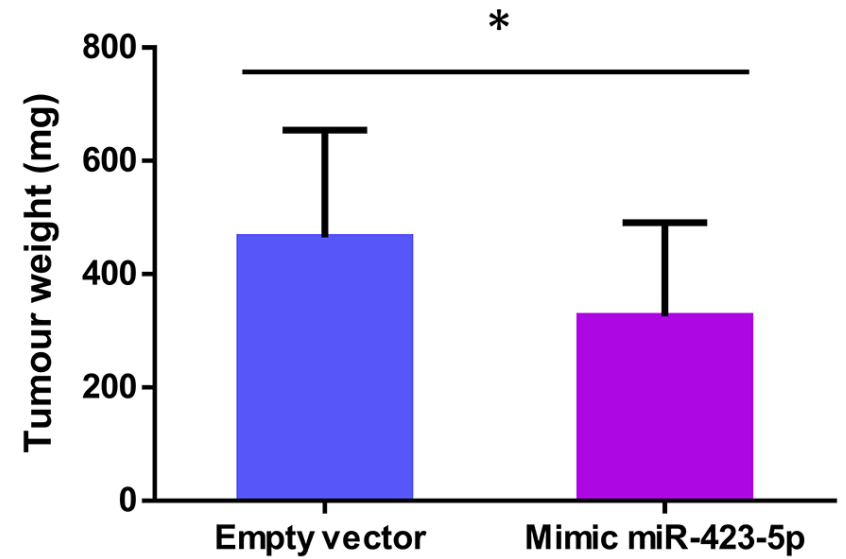

Supplement: Supplementary file 2 — Additional file 2: Additional figure 2. Surgically removed tumor tissues from nude mice of both control and miR-423-5p mimic group, at the end of the in vivo experiment and graphical representation of tumor weight in the different groups. Data are presented as the mean ± SD (n = 6, *p < 0.05). [file 13046_2021_2233_MOESM2_ESM.pdf]
